# Optical coherence tomography and elastography for *ex vivo* visualization of early gastric cancer

**DOI:** 10.1117/1.JBO.31.2.026501

**Published:** 2026-02-13

**Authors:** Alana G. Gonzales, Caitlin Ruhland, Graham Spicer, Stephen Mead, Massimiliano Di Pietro, Ashraf Sanduka, Photini F. S. Rice, Ryan H. W. Mitstifer, Sarah E. Bohndiek, Travis W. Sawyer, Jennifer Kehlet Barton

**Affiliations:** a University of Arizona, Wyant College of Optical Sciences, Tucson, Arizona, United States; b University of Arizona, Department of Biomedical Engineering, Tucson, Arizona, United States; c University of Cambridge, Department of Physics, Cambridge, United Kingdom; d Cancer Research UK Cambridge Institute, Cambridge, United Kingdom

**Keywords:** early cancer detection, optical coherence tomography, optical coherence elastography, gastric cancer

## Abstract

**Significance:**

Stomach (gastric) cancer survival depends significantly on the stage in which it is detected, and surveillance with white light endoscopy exhibits poor contrast between gastric cancer and healthy tissue, especially at early stages. Early gastric cancer can exhibit changes in epithelial microstructure, including loss of regular gastric pit structure and collagen alterations which increase tissue stiffness.

**Aim:**

To improve contrast between early cancer and normal tissue, we investigate the use of optical coherence tomography (OCT) and elastography (OCE) to visualize changes in tissue structure and stiffness consistent with gastric cancer.

**Approach:**

Images of eight samples of *ex vivo* human stomach tissue from three patients were collected with a benchtop OCT system. OCT was performed for qualitative visualization of tissue structure. OCE was then performed on 17 regions of interest using a simplified optical palpation method to extract relative stiffness measurements. A transparent silicone reference layer was placed on the tissue, and axial compression was applied. The resulting deformation (strain) of the reference layer was measured, and the corresponding stress applied to the sample surface was extracted from the characteristic stress-strain curve of the reference material. Spatially resolved stress measurements were mapped and overlaid on en face OCT images. Tissue classification was confirmed by pathology.

**Results:**

OCT image volumes showed more distinct gastric pit and tissue layer structure, as well as less optical attenuation, in normal tissue compared to gastric metaplasia and focal signet ring cell carcinoma (SRCC). Exemplary OCE-derived stress maps showed a trend of increasing measured stress with progression of precancer (metaplasia and dysplasia) and SRCC, suggesting increased tissue stiffness.

**Conclusions:**

This proof-of-concept study provides evidence that OCT and OCE may be capable of visualizing differences in tissue structure and stiffness between normal, metaplastic, dysplastic, and early cancerous gastric tissue, potentially providing the basis for improved screening tools with higher sensitivity.

## 1 Introduction

Stomach (gastric) cancer is the fifth most common cancer globally, with over one million new cases around the world each year^1^^,^^2^ and an estimated 30,300 new cases in the United States in 2025.^3^ There is an overall 36% 5-year survival rate, but survival depends significantly on the stage of cancer in which it is detected. Local gastric cancer has a 75% 5-year survival rate, whereas that rate drops to only 7% for distant cancer, which has spread to parts of the body remote from the primary tumor.^3^ Individuals at high risk of gastric cancer, such as those carrying a genetic variant in the tumor suppressor gene CDHI and with a family history of hereditary diffuse gastric cancer (HDGC), may be recommended to undergo risk-reducing total gastrectomy, which leads to long-term sequelae.^4^ A recommended alternative for individuals who decline or wish to postpone gastrectomy is yearly surveillance endoscopies with both targeted and random biopsies to detect microscopic foci of signet ring cell carcinoma (SRCC), which are characteristic of early HDGC.^4^^,^^5^ However, detection of early gastric cancer, including SRCC, is difficult using standard white-light endoscopic (WLE) examination. A skilled endoscopist and several targeted and random biopsies are required for reasonable sensitivity to early gastric cancer due to the poor contrast between gastric cancer and healthy tissue. For example, in HDGC patients undergoing endoscopic surveillance, the sensitivity of WLE-targeted biopsies alone can be as low as 42%.^5^ Thus, there is an unmet need for an accurate, rapid, cost-effective method of surveillance in patients at elevated risk for gastric cancer to detect it early and save lives.

### 1.1 Gastric Cancer and Optical Coherence Tomography

Early gastric cancer of various types is generally characterized by mucosal discoloration, morphological changes such as elevated, flat, or depressed structure, and changes in vascularity.^6^^–^^8^ Histologically, it exhibits changes in the normal epithelial microstructure, including loss of regular gastric pit structure, cell abnormalities, including signet ring cells, which are pathognomonic for diffuse type gastric cancer, and collagen alterations, which increase stiffness of the tissue.^8^^,^^9^ Visualization of these alterations may be challenging for white light imaging, but a new generation of advanced optical imaging modalities may be able to detect subtle tissue changes and potentially provide an effective method of gastric cancer surveillance.

Optical coherence tomography (OCT) is a nondestructive imaging modality that uses reflected near-infrared light to generate cross-sectional images of tissue with high resolution (∼10  μm) and depth of imaging up to 2 mm. The near-microscopic resolution and volumetric, subsurface imaging capabilities of OCT have driven its recent use in the gastrointestinal (GI) tract, especially applied to the surveillance of neoplastic progression toward esophageal adenocarcinoma (EAC) in Barrett’s Esophagus (BE) patients with endoscopic as well as capsule OCT systems.^10^^–^^12^ Endoscopic OCT has also been used for noninvasive evaluation of other areas of the GI tract such as the colon, the pancreatico-biliary tract, the small intestine, and the stomach, though fewer studies have focused on gastric lesions.^11^^,^^12^ It has been shown that endoscopic OCT can visualize changes in the tissue architecture associated with intestinal metaplasia and early gastric cancer such as irregular pit structure, absence of the layered structure of normal epithelium, disorganization with inhomogeneous tissue contrast, and increased vascularization.^13^^–^^15^ The use of endoscopic OCT in gastric cancer surveillance might provide improved sensitivity over WLE.

### 1.2 Optical Coherence Elastography

Optical coherence elastography (OCE) refers to a group of techniques that can measure and map mechanical properties of tissue, providing additional contrast between normal and diseased tissue.^16^^,^^17^ OCE has been used to characterize a wide range of biological tissue types and has previously been demonstrated as a promising method for delineation of soft-tissue tumors both *ex vivo* and intraoperatively.^18^ OCE is performed by subjecting a tissue sample to a mechanical load and imaging with OCT pre- and post-deformation.^16^ Using a mechanical model, the measured deformation is related to a mechanical property, such as elasticity (i.e., Young’s modulus).^16^ Different types of mechanical loading can be used, with the two most common methods being axial compression OCE (or just “compression OCE”) and transient wave OCE.^16^ Compression OCE works by applying a slow, quasi-static, compressive load across the surface of the sample,^16^^,^^19^ whereas transient OCE is a dynamic method that works by applying a pulsed load such as an air puff or laser pulse, which launches elastic waves.^16^^,^^17^ Various methods, such as speckle tracking, can then be used to measure tissue deformation. These methods can provide quantitative measures of tissue mechanical properties, but can be complex or computationally expensive.^16^

Our pilot study performs a simplified compression OCE method, referred to as optical palpation, on human stomach samples. The method is based on Kennedy et al., in which the deformation (strain) of a thin silicone reference layer with known mechanical properties overlaid on the tissue sample is measured from OCT volumes taken pre- and post-axial compression to evaluate relative tissue stiffness.^20^ In this method, the silicone reference layer, with stress-strain behavior known from calibration measurements, acts as an “optical sensor of stress.”^19^ Although the Young’s modulus of the tissue is not directly measured, optical palpation allows the calculation and mapping of the local stress at the sample surface from the measured strain of the reference layer. The required external stress to achieve a given reference layer strain is higher over stiff tissues, such as precancerous and cancerous tissues, compared to normal tissue.^16^^,^^20^^,^^21^

If tissue strain is measured with compression OCE while stress is measured with optical palpation, quantitative measures of tissue elasticity can be obtained using a method called quantitative micro-elastography (QME).^22^Allen et al. performed a comparison of optical palpation to QME that determined that elevated stress measured with optical palpation is consistent with elevated elasticity measured with QME when the applied compression is controlled.^21^ Clinically, relative measurements of tissue stiffness using estimation of applied stress can be sufficient for detecting abnormal regions of tissue using the simpler optical palpation method. Measuring stress directly with a force sensor can also enable quantitative measurements but is typically limited to point measurements, whereas optical palpation allows for spatially resolved stress measurements at the surface of the sample.^16^ Here, we demonstrate the feasibility of combined use of OCT and optical palpation OCE to visualize changes in structure and tissue stiffness between normal stomach tissue, metaplasia, dysplasia, and foci of SRCC in *ex vivo* human samples.

## 2 Materials and Methods

### 2.1 Gastric Tissue Samples

OCT and OCE were investigated as part of a clinical study on resected tissue specimens to evaluate novel imaging techniques for the detection of lesions in the upper gastrointestinal tract. Eight total specimens were resected from three patients at Cambridge University Hospitals, United Kingdom, either by endoscopic resection or surgical resection (gastrectomy). Both methods were deemed acceptable as all samples included the full mucosa and submucosa down to the muscle layer and were thicker than OCT imaging depth. In addition, samples were >5  mm in diameter and preserved tissue architecture. After imaging, specimens were returned to standard of care pathology, and gold-standard classification of samples was provided by histological sections obtained at ∼2  mm increments through the tissue. Regions of normal gastric mucosa, gastric metaplasia, focal intestinal metaplasia, focal low-grade dysplasia, focal high-grade dysplasia, and intramucosal SRCC were identified. Patients recruited for this study were aged 18 or over, clinically fit for a surgical procedure and had a previous confirmed diagnosis of gastric adenocarcinoma. Patients who had known coagulopathy, cardiopulmonary disease, or decompensated liver disease, or who had had a cerebrovascular event within the last 6 months, were excluded. The trial was reviewed by the Nottingham 2 Research Ethics Committee and was approved on 21 March 2018 (18/EM/0069). The trial was registered under IRAS ID 239559.

### 2.2 Silicone Reference Layer

For each sample, an optically transparent silicone reference layer approximately 600  μm thick was fabricated using a mixing ratio of 1:10:40 (Cross-linker: Catalyst: Polydimethylsiloxane (PDMS) oil). Wacker ELASTOSIL^®^ RT 601 parts A and B were used as cross-linker and catalyst, and MicroLubrol^®^ Type 200 Fluid Silicone Oil was used as PDMS oil. According to Lamouche et al.,^23^ this mixing ratio should provide a reference layer with an elastic modulus of 38 kPa, on the same order as that expected for most normal soft tissues.^24^ A sheet of material was cured at 90°C for 25 min, and a metal punch was used to create 10 mm diameter reference layers.

### 2.3 OCT Imaging

Within 1 h following resection of each of the eight tissue samples, images of the fresh specimens were collected with a commercial benchtop spectral domain OCT system (Thorlabs TEL320C1) and corresponding software (ThorImage OCT 5.5.5). System axial resolution was 5.5  μm in air, and the center wavelength was 1300 nm. An objective with lateral resolution of 13  μm was used (LSM03), and images were acquired at an A-scan rate of 76 kHz. Three-dimensional (3D) OCT images of each sample were obtained for qualitative visualization of the tissue architecture. Because some samples were larger than the maximum field of view (FOV) of the LSM03 objective (10 mm), and all had varying shapes, overlapping FOVs of 5 to 10 mm in the x- and y-directions (lateral) and 3.58 mm in the z-direction (depth) were obtained to visualize the entire sample. OCT volumes were 512 to 1024 pixels (lateral) × 1024 pixels (depth), depending on the FOV. Pixel size was 9 to 18  μm (lateral) × 3.50  μm (depth), which was sufficient spatial sampling to qualitatively assess each image.

### 2.4 OCE Imaging

Following OCT imaging of the entire sample, OCE was performed on individual FOVs of 5 to 10 mm in the x- and y-directions and 3.58 mm in the *z*-direction. The FOV was chosen to fit an area of the sample that was visually flat. Pixel size was 13.67  μm (lateral) × 3.50  μm (depth), and OCE images were acquired in a maximum of 5.7 s per image volume. For samples collected by endoscopic resection, one FOV was measured, whereas for samples collected by surgical resection, 2 to 4 FOVs were measured based on their larger size, resulting in a total of 17 OCE measurements across eight samples.

**Fig. 1:**
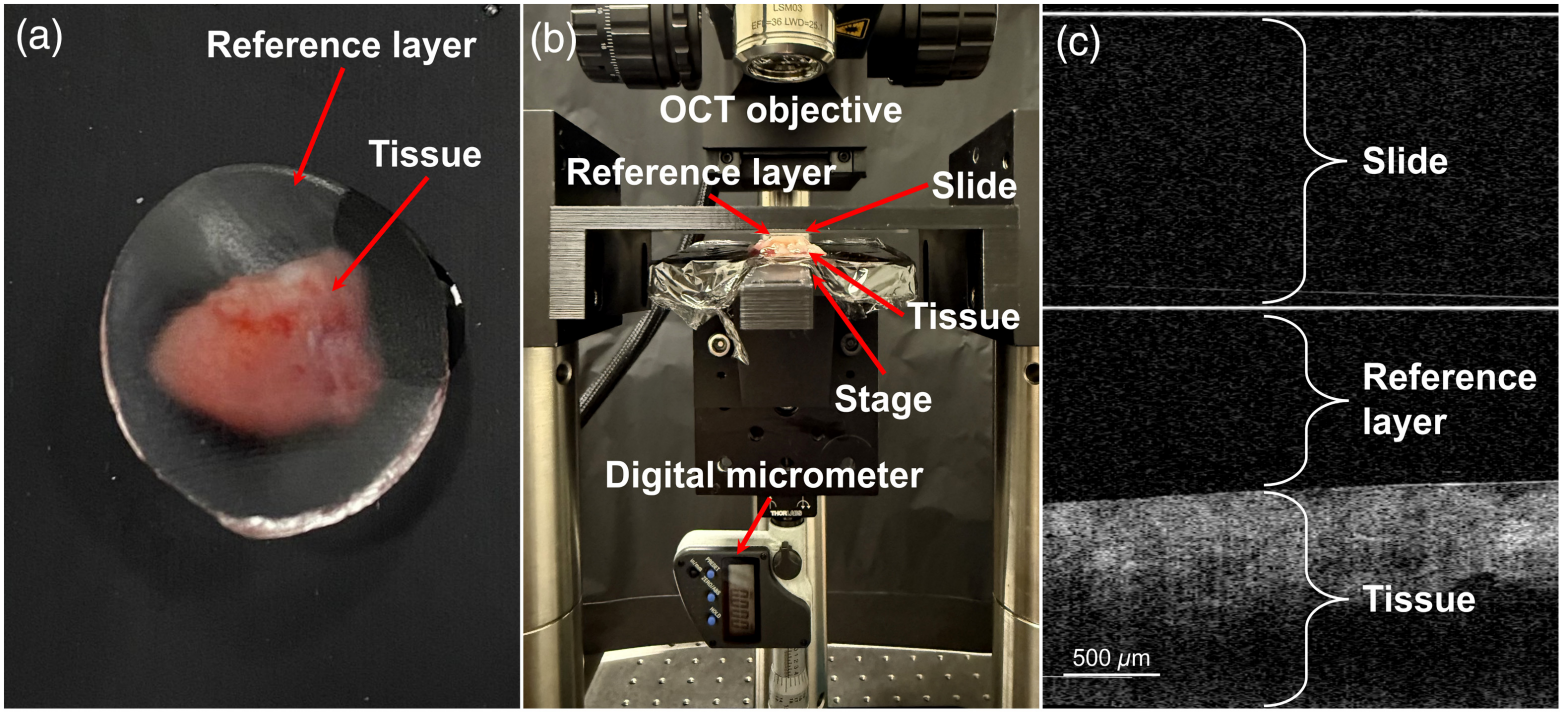
(a) Tissue sample with an overlaid reference layer. (b) OCE imaging setup showing tissue with an overlaid reference layer compressed together between a microscope slide and a mechanical stage controlled by a digital micrometer. (c) Example cross-sectional 2D slice of a 3D OCT image volume, showing visible layers of glass (microscope slide), silicone reference layer, and tissue.

The OCE setup is shown in Fig. 1. The tissue was placed on a *z*-direction translation stage, the silicone reference layer was placed on top, and the combination was translated until the reference layer was visually in contact with a stationary glass microscope slide mounted in a 3D-printed holder. The OCT objective imaged the tissue through the glass slide and reference layer. The stage was translated in the *z*-direction with precise 100  μm increments, measured using a digital micrometer, to axially compress the tissue and reference layer against the microscope slide. This method assumes, as is common with compression OCE methods, that the resulting applied stress was uniform in depth and that this stress was uniaxial. Therefore, the sample and the reference layer were assumed to be subjected to the same amount of stress, σ, applied by raising the stage. Previously reported optical palpation methods^20^^,^^21^ utilized only two image volumes, taken at a preload condition (with the tissue slightly compressed in firm contact with the stage and reference layer) and a compressed condition (after a consistent stage movement). Variation in the preload condition could impact the ability to make comparisons across samples. In our work, up to 16 OCT image volumes were collected as the stage was raised in 100  μm increments. The initial stage position of visual contact may have provided insufficient physical contact between the reference layer and tissue, or excessive fluid may have been present at the interface; therefore, little reference layer strain occurred with initial stage movement. We defined the preload condition as the stage position where subsequent movement caused a distinct and linear decrease in reference layer thickness. The compressed condition was three-stage increments (300  μm movement) after the preload.

### 2.5 OCE Data Analysis

#### 2.5.1 Reference layer strain measurement

In the linear regime, where applied stress, σ, linearly affects strain, ε, the elastic modulus is the Young’s modulus, E, which follows the relationship in Eq. (1),

σ=Eε. (1)

This simplified optical palpation method operated under the assumptions that the stress applied to the reference layer ($\sigma_{1}$) was uniform in depth, uniaxial, and equal to the stress applied to the tissue sample ($\sigma_{2}$). This resulted in a direct relationship between the applied stress on both the tissue and reference layer (σ) and the Young’s modulus of the sample ($E_{2}$) given by Eq. (2),

$$\sigma =\sigma_{1}=\sigma_{2}=E_{1}\varepsilon_{1}=E_{2}\varepsilon_{2}.$$ (2)

In addition, it was assumed that the tissue sample and reference layer were each homogeneous in depth. Thus, one-dimensional strain of the reference layer along the axis of compression (axial strain, $\varepsilon_{1}$) could be calculated by Eq. (3), where ΔL was the change in reference layer thickness between preload and compressed states, and *L* was the preload reference layer thickness,

$$\varepsilon_{1}=\frac{\Delta L}{L}.$$ (3)

For each sample, OCT volumes were visually assessed and cropped to isolate regions of interest that were as flat as possible (within 14% surface height variation), were at least 1 mm from the edge of the sample, had no reflection artifacts brighter than the axial borders of the reference layer (glass-reference layer and reference layer-tissue interfaces), and maintained reference layer-tissue contact in all OCT image volumes with increasing stage displacement. The thickness of the reference layer was then measured at every A-scan in the cropped OCT image volume. This was done in MATLAB by finding the two highest maxima in each A-scan, which largely corresponded to the axial borders of the reference layer based on the principles of Fresnel reflection. The distance between these two maxima was calculated, which provided the reference layer thickness in pixels. When automatically finding the maxima, the tolerance for peak prominence was set to exclude measurements where the two highest maxima were not prominent enough to indicate borders of the reference layer. Average reference layer thickness across each OCT volume was then plotted versus stage displacement. For each pixel coordinate in the *xy*-plane across the OCT volumes, ΔL was calculated as the change in reference layer thickness between the preload and compressed states. Strain of the reference layer (unitless) at each *xy*-point was calculated using Eq. (3).

#### 2.5.2 Reference layer characterization and stress calculation

Independent measurements of the silicone reference layer were performed to assess its stress-strain behavior. Three reference layers were measured using the same OCE setup shown in Fig. 1, except that a SingleTact miniature force sensor was placed on top of the translation stage, beneath the reference layer to directly measure the force applied under axial compression. The stage was translated in the *z*-direction in increments of 100  μm until the force measurement exceeded the range of the force sensor (4.5 N). At each increment, an OCT image and a force measurement were collected, then the compressed diameter and the average thickness of the reference layer were measured from the OCT image. Stress on the reference layer was calculated as the measured force divided by the area of the reference layer, and strain was calculated as the change in thickness divided by the original thickness. Stress was plotted as a function of strain of the reference layer calculated by Eq. (2), each data point representing the stress and strain measurements obtained for a consecutive increment of stage displacement. Linear regression analysis was performed in the low-strain region, and the Young’s modulus of the material was extracted from the slope of this line.

Local tissue stress values corresponding to the local strain values were obtained from this stress-strain curve of the reference material (all were in the linear low-strain regions). Maps of the applied stress on the tissue samples were generated in MATLAB by converting the two-dimensional matrix of stress values for each OCT volume to a colormap. A Gaussian blur with a standard deviation of 3 pixels was applied to each stress map to smooth small discontinuities from occasional erroneous peak detection. A Fourier band-stop filter was applied to reduce discretization noise caused by the finite *z*-pixel size. Stress maps were then overlaid on corresponding top-down OCT images of tissue. The average value of stress for each map was also computed. Based on Eq. (2), stress maps reflect the relative measurements of tissue stiffness.

ChatGPT (OpenAI, Inc., version 4o) was utilized as an image analysis code writing assistant to analyze the displacement of the reference layer from OCT images. Prompts directed the translation of an ImageJ (version 1.54p, open source) macro into MATLAB (MathWorks, Inc., version R2023a) code. Additional prompts were used to refine the code, which included producing stress maps and average stress measurements. Generated code was checked by an experienced MATLAB programmer, and spot comparisons of measurements were made against manually calculated results.

## 3 Results

### 3.1 OCT Images Show Less Distinct Gastric Pit Structure and Greater Attenuation in Abnormal Compared with Normal Gastric Mucosa

**Fig. 2:**
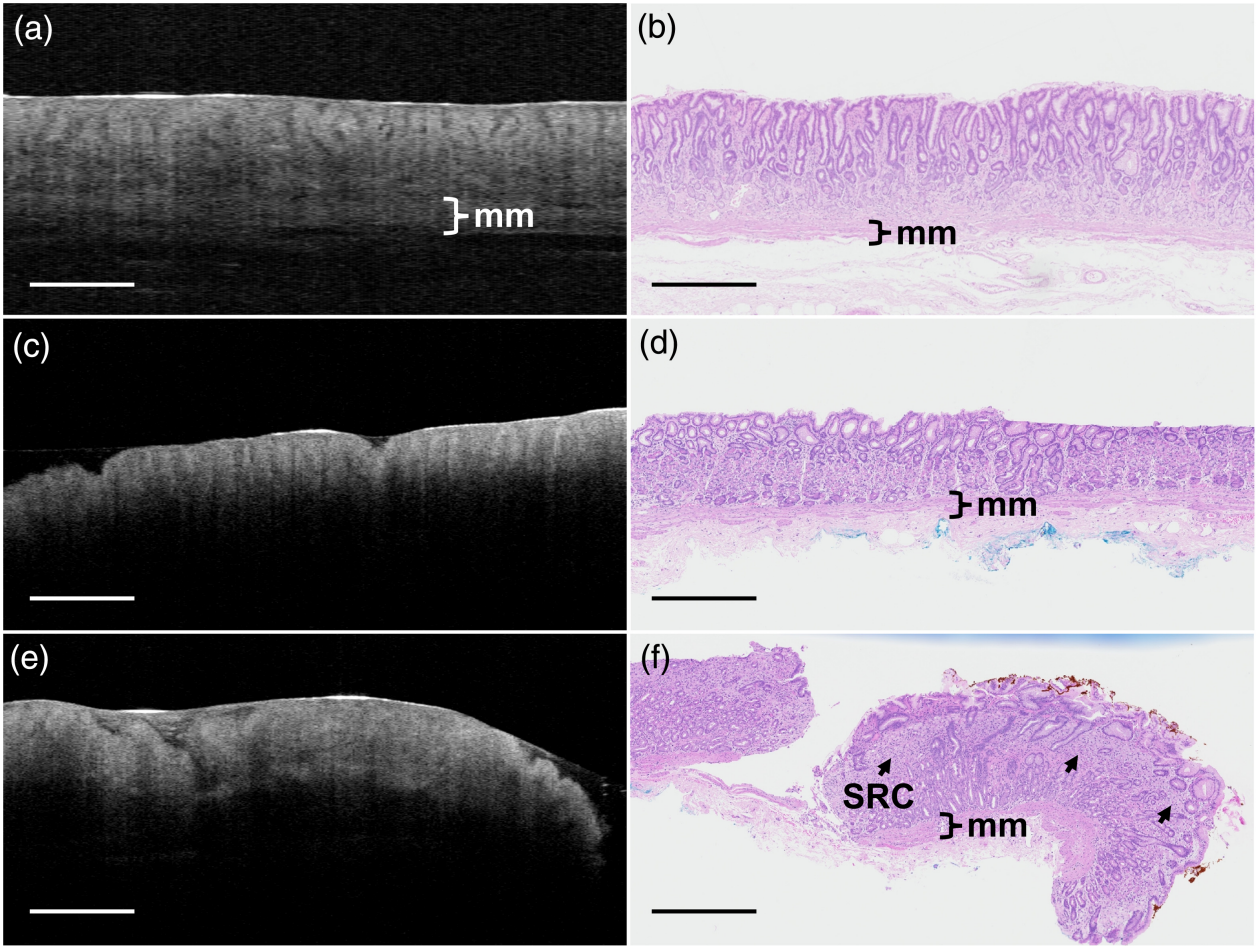
Cross-sectional OCT images (left) and corresponding H&E-stained histology sections (right) of normal gastric mucosa (a, b), gastric metaplasia (c, d), and intramucosal SRCC (e, f). The muscularis mucosa (mm), evident in histology, appears attenuated in OCT images of metaplasia and SRCC. Focal clusters of signet ring cells (SRC) are visible in the histology of SRCC, manifesting as a more homogeneous appearance of the mucosa in the OCT image. For all images, the field of view is 3 mm (width) × 1.5 mm (depth), and all scale bars are 500  μm.

Example cross-sectional OCT images of normal gastric mucosa, gastric metaplasia, and intramucosal SRCC are shown in Figs. 2(a), 2(c), and 2(e), respectively. All images are cropped to 3 mm in width and 1.5 mm in depth to highlight areas of interest. Corresponding H&E-stained histology images of the same samples are shown in Figs. 2(b), 2(d), and 2(f), respectively. Qualitative analysis of OCT images shows more distinct gastric pit structure and tissue layer structure in normal gastric mucosa compared with gastric metaplasia and intramucosal SRCC, consistent with observations by Poneros et al.^15^ There is also noticeably higher attenuation of the OCT signal with depth in both metaplasia and SRCC, masking the underlying muscularis mucosa, compared to normal tissue. Histology images show that in gastric metaplasia and SRCC, the structure of the mucosa is visibly less organized than normal gastric mucosa, with greater variability in gastric pit size and shape. Further, the histology image of intramucosal SRCC shows dense regions of signet ring cells in the mucosa, which disrupt the normal pit structure. These signet ring cells are not directly visible in the OCT image but manifest as a more homogenous appearance of the mucosa, with less visible pit structure.

### 3.2 Example Thickness Data and Stress-Strain Curve of Silicone Reference Material

**Fig. 3:**
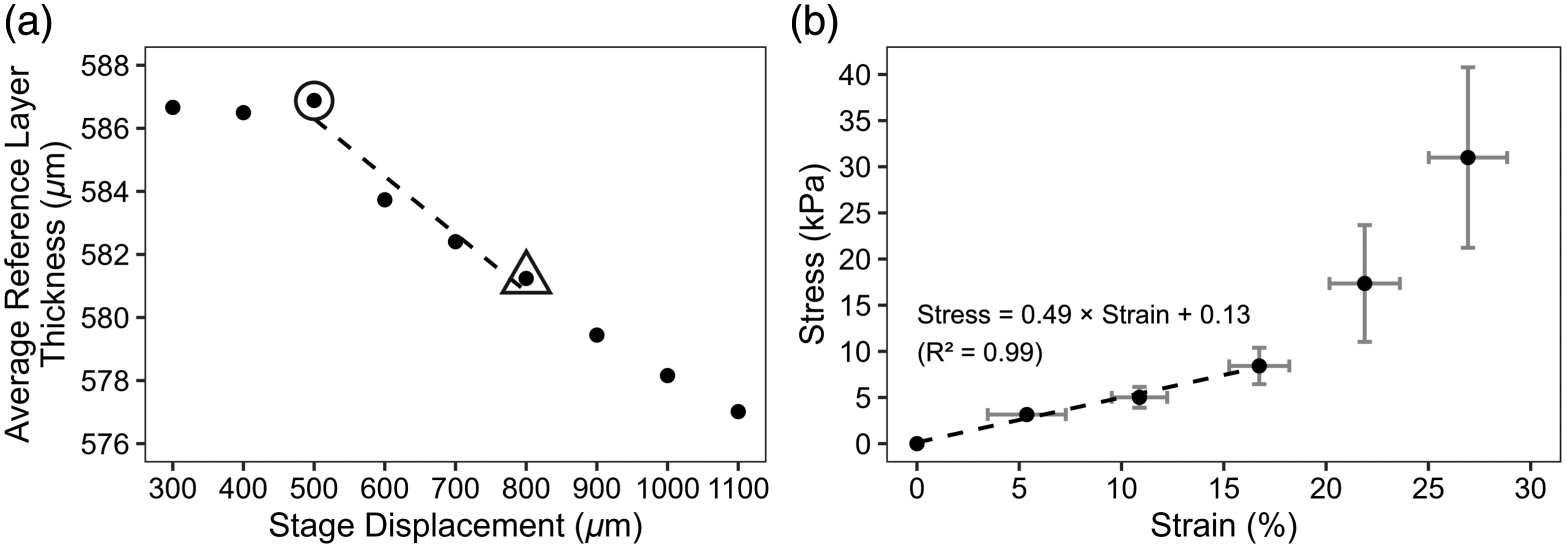
Example average reference layer thickness data as a function of *z*-stage movement (a), showing initial lack of thickness change, then a linear decrease. The circled data point indicates the preload state, and the data point inside a triangle represents the compressed state. The dashed line shows the approximately linear portion of the data corresponding to 300  μm of *z*-stage displacement over which strain was calculated. Conversion to stress was accomplished by mapping to the calibration stress-strain curve of the reference material (b) (n=3, mean ± *SD*). Each data point is a 100  μm *z*-stage movement. A linear fit to the low strain region (line of best fit, $R^{2}=0.99$) yields a slope of 0.49, corresponding to an elastic modulus of ∼49  kPa.

An example data set of average reference layer thickness as a function of z-stage displacement is shown in Fig. 3(a). The first data points show little change, due to insufficient physical contact between the sample and reference layer. The subsequent data points show a linear decrease in reference layer thickness with stage movement. The circled data point (the “preload” state) and the data point inside a triangle (the “compressed” state) were used for subsequent calculations. The approximately linear fit over this region is shown by a dashed line.

Figure 3(b) shows the characteristic stress-strain curve obtained from independent measurements of the reference layer material as an average of three replicates (mean ± *SD*), each data point corresponding to a consecutive 100  μm increment of *z*-stage movement. The elastic modulus of the reference layer calculated as the slope of the stress-strain curve in the regime of low strain, shown by the dashed line, as discussed by Lamouche et al.,^23^ is ∼49  kPa.

### 3.3 OCE Data Shows Higher Stress Measured for Abnormal Compared with Normal Gastric Mucosa

**Fig. 4:**
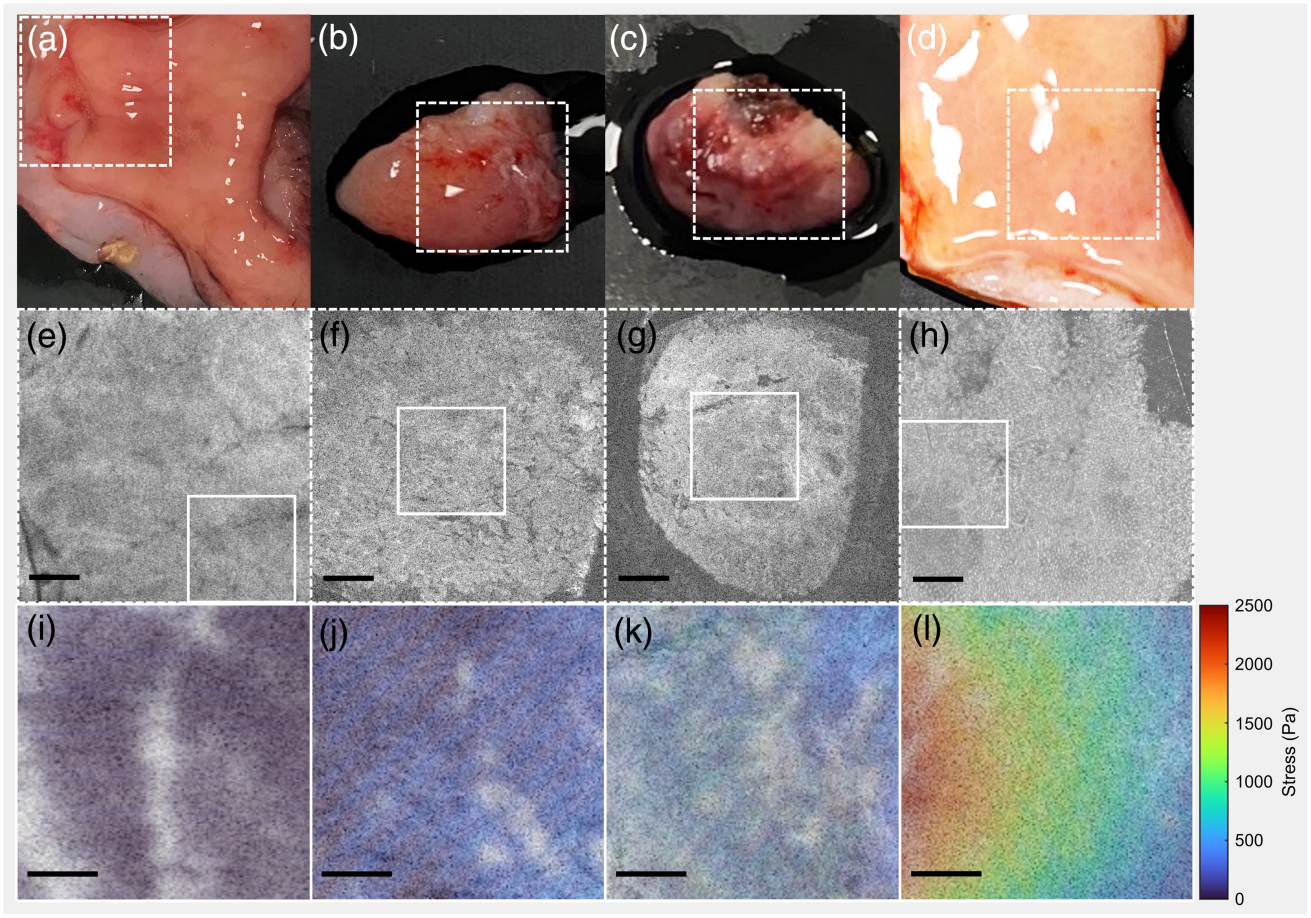
Photographs of *ex vivo* samples of human stomach tissue (a–d) collected from patients either by gastrectomy (a and d) or endoscopic mucosal resection (b and c) showing the 6×6  mm lateral FOV of the OCT image volumes in the dashed-line boxes. The second row (e–h) shows en face OCT images, with areas where OCE data was analyzed identified by solid-line boxes (2.2×2.2  mm), scale bars are 1.0 mm. Corresponding stress maps are shown in the third row (i–l); scale bars are 0.5 mm. From left to right column, tissue is: a/e/i normal gastric mucosa, b/f/j focal gastric metaplasia and intestinal metaplasia, c/g/k focal low- and high-grade dysplasia, and d/h/l multiple foci of intramucosal SRCC.

Example photographs, OCT images, and stress maps generated from OCE data for four classifications of tissue are shown in Fig. 4. Photographs of the tissue are shown in Figs. 4(a)–4(d), with the FOV of the OCT image volume in the dashed-line box. An en face slice from the OCT image volume is given in Figs. 4(e)–4(h). All OCT images have been cropped to match the size of the smallest FOV of the four OCT images, which was 6 mm in the *x*- and *y*-dimensions. These OCT image slices show regions of interest where stress was measured in solid-line boxes. The stress maps are shown in Figs. 4(i)–4(l). All stress maps have been cropped to match the size of the smallest FOV of the four images, which was 2.2 mm in the *x*- and *y*-dimensions.

Columns correspond to tissue sample-level diagnosis: from left to right, the samples contain normal gastric mucosa [Figs. 4(a), 4(e), 4(i)], regions of gastric metaplasia and focal intestinal metaplasia [Figs. 4(b), 4(f), 4(j)], focal low- and high-grade dysplasia [Figs. 4(c), 4(g), 4(k)], and focal intramucosal SRCC [Figs. 4(d), 4(h), 4(l)], as verified with spatially corresponding pathology results. There is a visual increase in average measured stress with the progression from normal tissue to metaplasia and dysplasia to SRCC, which suggests an increase in tissue stiffness. Regions of the maps where there is no stress information appear light gray. This situation occurred where image artifacts prevented an accurate measurement. The region shown in the stress map was chosen because that area was optimal for performing measurements of stress (flat and good reference layer-tissue contact) and because there was corresponding spatial pathology available.

**Fig. 5:**
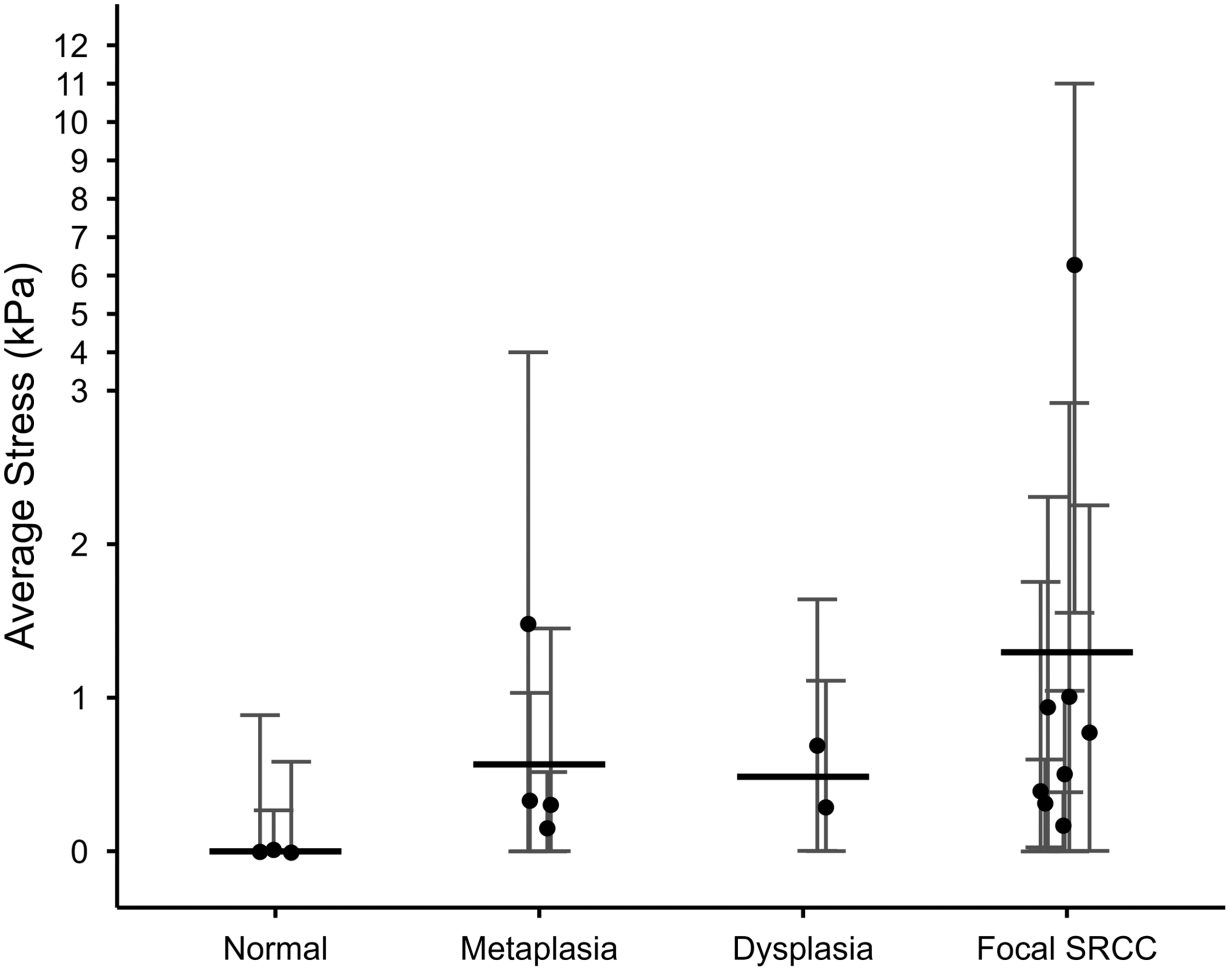
Average stress (mean ± *SD*) calculated across each of 17 FOVs imaged with OCE, grouped by sample classification [normal (n=3), metaplasia (n=4), dysplasia (n=2), or focal SRCC (n=8)], shown as black solid circles. Solid bars represent the overall mean for each group.

A summary of all 17 average stress measurements collected via OCE and categorized by sample diagnosis is shown in Fig. 5. Three measurements were taken from one sample classified as normal, four measurements were taken from three samples classified as gastric metaplasia and/or focal intestinal metaplasia, two measurements were taken from two samples classified as focal low- and/or high-grade dysplasia, and eight measurements were taken from two samples classified as focal intramucosal SRCC. Each OCE measurement is plotted as a solid circle, and the mean average stress for each group is shown as a solid bar. One focal SRCC sample is a high-stress outlier; histology of that area reveals a focus of SRCC that is about three times as large as any other sample.

## 4 Discussion and Conclusion

In this proof-of-concept study, we saw evidence that OCT is capable of visualizing changes in tissue structure consistent with gastric metaplasia and foci of intramucosal SRCC in *ex vivo* human stomach samples. Images of normal tissue showed more distinct gastric pit structure and tissue layer structure compared to images of metaplasia and SRCC foci. This finding is consistent with previous literature.^13^^–^^15^ We also observed an increase in attenuation of the OCT signal in metaplasia and SRCC foci, likely due to changes in tissue organization and, in the case of SRCC, the presence of densely packed signet ring cells disrupting the normal pit structure. Further, we saw evidence that OCE, using this simplified optical palpation method, may be capable of visualizing variations in tissue stiffness by mapping the local stress at the sample surface under axial compression, measured via a reference layer. In contrast to previous optical palpation methods that measure reference layer deformation using only two (pre- and post-compression) images,^20^^,^^21^ we obtained multiple measurements at increasing amounts of compression to more precisely establish the preload condition. This ensures that the compressed state was within the linear regime of material deformation. This method may allow better comparison between samples.

Comparing samples, exemplary results showed a progression of stiffness from normal to focal SRCC. When all 17 OCE measurements were plotted by sample diagnosis, our samples of normal tissue showed lower stiffness than diseased tissue, although there is no significant difference between groups due to the low sample number. Our findings are consistent with published literature on optical palpation OCE in similar tissue types, such as cancer of the breast, where invasive tumors had increased tissue stiffness compared with adipose tissue and benign dense tissue.^21^ Although a phase-resolved variant of compression OCE has been used to show increased stiffness in clusters of signet ring cells in the colon,^25^ we are the first, to our knowledge, to evaluate gastric SRCC with optical palpation OCE.

White light surface imaging of gastric tissue has limited sensitivity for these early stages of gastric cancer. We demonstrate the feasibility of the relatively simple and clinically translatable technologies of OCT and optical palpation OCE to visualize changes in tissue microstructure and stiffness associated with gastric disease. The major limiting factor of this study was the small sample size, including limitations to only one normal specimen, from which we took three OCE measurements, and further studies will need to be performed to validate our findings. A challenge in this study was the heterogeneity of the samples and focal nature of the disease. For example, a sample containing foci of SRCC also contained regions of normal gastric mucosa and gastric metaplasia. Since spatial pathology was provided, we know the mix of diagnoses in the OCT images and stress maps shown. Histology showed that regions of metaplasia and dysplasia involved areas between 1 and 20 mm square, and regions of SRCC involved areas between 1 and 5 mm square. For purposes of analysis, the most severe diagnosis was applied to the sample. Therefore, it is unsurprising that the diseased samples showed a large variation in stress, which can be seen in the error bars in Fig. 5. Diseased regions were on the order of the optical palpation imaging area, and the heterogeneities were larger than the system’s resolution.

Another challenge of this work was tissue surface unevenness. Sanderson et al. examined this deviation from the flat-surface assumption and found that surface roughness affected sensitivity but had a relatively small effect on the accuracy of elasticity measured by QME.^26^ Our tissue samples had a surface height variation of up to 14% in regions analyzed with OCE, so while surface unevenness likely affects the results, the data suggest that the differences in stiffness between normal and abnormal tissues can still be identified. The data presented in Fig. 5 are presented as average stress over each region of interest; averaging likely also mitigates the effects of tissue surface unevenness.

The sensitivity of stress measurements is dependent on the mechanical properties of the reference layer. The elastic modulus of our reference material was measured to be 49 kPa, higher than predicted based on formulation^23^ and higher than most normal soft tissues. Thus, stress measurements near zero were seen for reference layers overlaid on normal gastric tissue. Nonetheless, our OCE system provided sufficient sensitivity to detect differences between normal and abnormal tissues. Lastly, the assumption in the optical palpation method that stress is only applied uniaxially and there is negligible friction between the glass slide, reference layer, and tissue can result in errors in stress measurements, especially for small amounts of reference layer displacement.

### 4.1 Future Directions for Research

Future work aims to investigate the use of this method on a larger sample size of *ex vivo* patient samples. For clinical translation, a forward-facing endoscope could be developed for regular surveillance of individuals at high risk of gastric cancer. Conventional gastroscopes commonly have a 2.8 mm-diameter working channel, and an endoscope with an outer diameter around 2.5 mm could be inserted through such a working channel during regular endoscopy for easy implementation in the screening process. Optical palpation could be implemented with a thin compressible layer at the distal tip. Lastly, increased tissue stiffness is a common characteristic of several cancer types, making this technique potentially applicable to the detection of other cancers.

## Data Availability

Code for analyzing OCE data is available on GitHub under the username “alanagonzales-ua” in the public repository titled “oct-elastography” (https://github.com/alanagonzales-ua/oct-elastography). All data in support of the findings of this paper are either available within the article or are available in the University of Arizona Research Data Repository.
